# LINCing Senescence and Nuclear Envelope Changes

**DOI:** 10.3390/cells11111787

**Published:** 2022-05-30

**Authors:** Bakhita R. M. Meqbel, Matilde Gomes, Amr Omer, Imed E. Gallouzi, Henning F. Horn

**Affiliations:** 1 College of Health and Life Sciences, Hamad Bin Khalifa University, Doha P.O. Box 34110, Qatar; bakmeqbel@hbku.edu.qa; 2 KAUST Smart-Health Initiative and Biological and Environmental Science and Engineering (BESE) Division, King Abdullah University of Science and Technology (KAUST), Jeddah 21589, Saudi Arabia; matilde.gomes@kaust.edu.sa (M.G.); gallouzi.imed@kaust.edu.sa (I.E.G.); 3 Department of Biochemistry, McGill University, 3655 Promenade Sir William Osler, Montreal, QC H3G 1Y6, Canada; amr.omer@mail.mcgill.ca

**Keywords:** senescence, LINC complex, Nesprins, SUN, nuclear envelope

## Abstract

The nuclear envelope (NE) has emerged as a nexus for cellular organization, signaling, and survival. Beyond its role as a barrier to separate the nucleoplasm from the cytoplasm, the NE’s role in supporting and maintaining a myriad of other functions has made it a target of study in many cellular processes, including senescence. The nucleus undergoes dramatic changes in senescence, many of which are driven by changes in the NE. Indeed, Lamin B1, a key NE protein that is consistently downregulated in senescence, has become a marker for senescence. Other NE proteins have also been shown to play a role in senescence, including LINC (linker of nucleoskeleton and cytoskeleton) complex proteins. LINC complexes span the NE, forming physical connections between the cytoplasm to the nucleoplasm. In this way, they integrate nuclear and cytoplasmic mechanical signals and are essential not only for a variety of cellular functions but are needed for cell survival. However, LINC complex proteins have been shown to have a myriad of functions in addition to forming a LINC complex, often existing as nucleoplasmic or cytoplasmic soluble proteins in a variety of isoforms. Some of these proteins have now been shown to play important roles in DNA repair, cell signaling, and nuclear shape regulation, all of which are important in senescence. This review will focus on some of these roles and highlight the importance of LINC complex proteins in senescence.

## 1. Introduction

### 1.1. Senescence

Senescence is a highly dynamic and multi-step process that is essential for embryonic development but also contributes to regeneration and homeostasis in adulthood [1,2,3,4,5,6,7,8]. Senescence is fundamentally an irreversible growth arrest that can be triggered through intra- or extracellular stimuli. In general, senescence is associated with some of the detrimental aspects of aging, such as loss of skin elasticity and increased incidence of cancer; however, in some scenarios, senescence can also contribute to healing [9].

A variety of stresses are known to induce cellular senescence, including cell-division-associated telomere shortening (replicative senescence) [10,11,12], telomere dysfunction (telomere uncapping) [13], mitochondrial deterioration [14], oxidative stress [15,16,17], topoisomerase inhibitors (anticancer agents) [18,19,20], severe or permanent DNA damage and chromatin disruption [21] (genotoxic stress), as well as oncogene activation (oncogene-induced senescence). These stresses trigger the activation of p53–p21 and/or p16INK4a-pRB tumor suppressor pathways, which are two key pathways that promote proliferation arrest in senescence [12,22].

Senescence is an important event that drives tissue fate. Senescent cells affect the cellular environment through two potential mechanisms, cell autonomous and non-cell autonomous. Cell autonomous is restricted to the senescent cell and is centered on the inability to proliferate and contribute to tissue expansion/healing. The non-cell autonomous mechanism is associated with changes in proteins secreted by the cell and include inflammatory cytokines and proteases. These secreted proteins are collectively referred to as SASP (senescence-associated secretory phenotypes) and impact the cellular environment around the senescent cell. The secretion of SASPs attract and activate innate and adaptive immune cells, such as natural killer (NK) and T cells, normally inducing immune clearance of senescent cells [23]. With age and in some disease states, the immune system can become less effective in clearing senescent cells, leading to an accumulation of senescent cells that can be a significant source of SASPs [4,5,24,25,26]. While SASPs can promote tissue repair and regeneration [2,3], SASPs can also promote tissue dysfunction as a result of chronic inflammation, aging and age-related diseases, such as cancer [27,28,29].

Interestingly, senescent cells exhibit a series of distinct changes. Senescent cells are known to be larger, with a flattened appearance and increase in cytoplasmic granularity [30,31,32]. The nucleus in senescent cells is also larger and undergoes distinctive senescence-induced changes, including changes in nuclear lamina proteins. Indeed, the loss of Lamin B1 has become a marker for senescent cells [33,34,35,36]. Furthermore, mutations in Lamin A/C, another key component of the nuclear lamina, lead to premature aging phenotypes in mice and humans. The premature aging phenotypes are associated with a significant population of senescent cells [37,38]. The nuclear lamins clearly play an important role in senescence. However, other NE components have emerged as having a role in senescence and this review we will focus on LINC complex proteins and discuss their role in senescence (Table 1).

### 1.2. The LINC Complex

**Figure 1:**
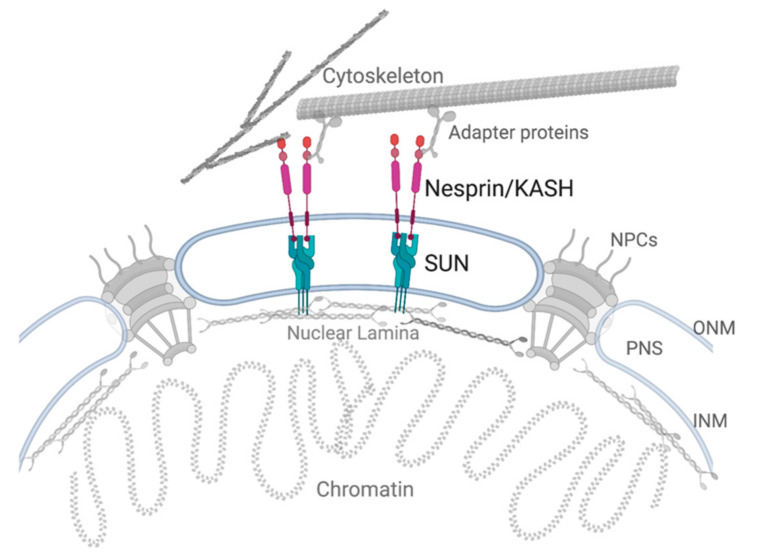
The LINC complex spans the nuclear membrane and connects the cytoskeleton to the nucleoskeleton. The nuclear membrane is composed of an outer nuclear membrane (ONM) and inner nuclear membrane (INM), which are separated by a perinuclear space (PNS). The ONM and INM are connect at sites of nuclear pore complex (NPC) insertion. SUN-domain proteins reside in the INM and connect to ONM KASH-domain proteins in the PNS, forming the core of the LINC complex. KASH domain proteins connect to the cytoskeleton either by direct interaction (actin) or through adapter proteins (microtubules (shown) and intermediate filaments (not shown)). Created with BioRender.com (accessed on 20 May 2022).

The nucleus is surrounded by a double membrane system that forms part of the nuclear envelope (NE) and serves, in part, to separate the nucleoplasm from the cytoplasm. The inner nuclear membrane (INM) sits adjacent to the nuclear lamina, a network of intermediate filament proteins that contribute to the mechanical resilience and structure of the nucleus (recently reviewed in [39]) and that play an important role in aging and senescence [40]. The outer nuclear membrane (ONM) is a continuous membrane system with the endoplasmic reticulum. The two membranes are separated by a lumen known as the perinuclear space (PNS) but connect to each other at sites of nuclear pore insertion. The ONM and INM are host to a number of transmembrane proteins that give functionality to the nuclear envelope and allow the nucleus (and the cell) to respond to a myriad of intra-and extracellular cues. An important family of protein complexes that spans both membranes and connect the nucleoskeleton with the cytoskeleton are the LINC (linker of nucleoskeleton and cytoskeleton) complexes (Figure 1). The complexes comprise of an INM SUN-domain protein and an ONM KASH-domain protein (recently reviewed in [41]). The respective SUN and KASH domains of the eponymous proteins form physical connections in the PNS, which allow for the direct transmission of mechanical strain from the cytoskeleton to the nucleoskeleton. Six KASH domain proteins have been identified, Nesprins-1, -2, -3, -4, KASH5 and LRMP [42]. All except LRMP have been shown to interact with the cytoskeleton. Nesprins-1 and -2 interact with the actin cytoskeleton via N-terminal actin binding domains. They have also been shown to interact with microtubules via kinesin and dynein binding domains [43]. Nesprin-3 interacts with intermediate filaments via the adapter protein plectin [44]. Nesprin-4 interacts with the motor protein kinesin and consequently, with the microtubule network [45]. Finally, KASH5 binds to dynein and through this motor, it interacts with the microtubule network [46,47].

These KASH-domain proteins are single-pass transmembrane proteins that reside in the ONM. The N-terminus is cytoplasmic, while the C-terminal KASH domain juts into the PNS. In the PNS, the KASH domain interacts with the SUN domain of INM SUN-domain proteins. SUN-domain proteins are INM transmembrane proteins that are essential binding partners for the ONM localization of KASH-domain proteins. The formation of a functional LINC complex through the physical binding of the SUN and KASH domains is essential for many of the functions of LINC complexes [42], including nuclear positioning [57] and nuclear shape and size [58,59]. In addition, alternate roles of KASH domain proteins have also been described, independent of their roles in LINC complexes [50,60,61,62,63,64]. In this review, we will discuss both LINC-dependent and LINC-independent roles for SUN and KASH domain proteins in the context of senescence.

## 2. LINC Proteins in Senescence

### 2.1. Nesprin-1

Nesprin-1 is the largest KASH-domain protein, with the full-length protein having a size of over 1 mega Dalton. Nesprin-1 links the nucleus to the actin cytoskeleton and is vital for nuclear positioning and regulating nuclear size [65,66]. A large number of smaller Nesprin-1 isoforms can be generated through alternate splicing and alternate transcriptional start sites [60], some of which do not contain the KASH domain and are consequently not membrane bound [61,62]. Some of these smaller isoforms are expressed in tissue-specific manners, and some are found on the inside of the nuclear membrane [63]. Nesprin-1, therefore, has the potential for a range of functions through these isoforms and has recently been shown to have roles in the process of senescence.

Radiation-induced senescence is triggered by doses of radiation that cause non-reparable DNA damage [67,68]. In the breast cancer cell line MCF7 and osteosarcoma cell line U2OS, γ-irradiation not only caused detectable DNA damage 24 h post-irradiation, but around 60% of cells exhibited nuclear morphology changes, such as micronuclei, invaginations, blistering and fragmentation. Seven days after irradiation, the majority of the cells are senescent and display intense nuclear morphology changes, as well as being positive for SA-β-galactosidase (SA-β-gal) [69]. Two Nesprin-1 isoforms, one at 150 kDa, the other at 250 kDa, were found to be increased in MCF-7 cells exposed to γ-irradiation. This increase started at 48 h after irradiation and continued for seven days. Interestingly, U2OS cells did not show a comparable increase in the 250 kDa Nesprin-1, instead the 250 kDa isoform of Nesprin-1 remained constant, the 150 kDa isoform disappeared and a new 125 kDa isoform appeared in senescent cells [69]. These changes were also accompanied by a localization of Nesprin-1 into micronuclei and cytoplasmic aggregates that were formed in response to the γ-irradiation [69]. The mechanisms behind the generation of these different Nesprin-1 isoforms are not known. However, protein cleavage has been suggested as the most likely mechanism [69]. Regardless of how these smaller species of Nesprin-1 are generated, it appears from the above observations that changes in Nesprin-1 localization and expression are associated with radiation-induced senescence.

Not only is radiation-induced senescence associated with changes in Nesprin-1, but a knockdown of Nesprin-1 led to senescence in a number of cell types. When Nesprin-1 was knocked down in non-transformed human fibroblasts, mouse fibroblast cell line (C3H10T1/2) and human normal liver epithelial cells (THLE-2), it caused a significant increase in the number of SA-β-gal positive cells. In addition, these cells had enlarged and often deformed nuclei consistent with senescent cells (i.e., folds, lobulations, protrusions, and micronuclei) [48,49]. How Nesprin-1 contributes to these senescence-associated changes in nuclear morphology has not been described. However, it has been shown that Nesprin-1 and Nesprin-2 are able to interact with Nesprin-3 at the NE and regulate nuclear size, with the increased expression of these Nesprins leading to smaller nuclei [59]. It is, therefore, possible that the loss of Nesprin-1 promotes senescence-associated nuclear changes through the loss of key interactions that normally regulate nuclear shape.

However, Nesprin-1 appears to play a role in senescence beyond mediating nuclear morphology changes. Persistent DNA damage is a key trigger that drives cells to enter senescence [70,71]. Nesprin-1 has been shown to interact with the DDR (DNA damage repair) machinery at several levels, including interactions with the DNA mismatch repair proteins MSH2 and MSH6, as well as the NHEJ repair machinery [49]. Using the hepatocellular carcinoma cell lines Hep3B and Huh7, it was found that these cells not only express low levels of Nesprin-1 but also have elevated basal levels of DNA damage (as measured by γH2AX staining), compared to normal liver cells [49]. When Nesprin-1 was knocked down in normal liver cells, this led to an increased accumulation of γH2AX staining. Conversely, when Nesprin-1 was ectopically expressed in the Hep3B and Huh7 cells, the level of DNA damage was decreased [48]. A knock-down of Nesprin-1 in the mouse embryonic mesenchymal stem cell line (CH310T1/2) and in human dermal fibroblasts (HFs) also led to an increase in DNA double strand breaks [49]. These findings are also consistent with observations in *S. pombe*, where the Nesprin-1 orthologue Kms1 was shown to be important for the repair of double strand breaks [72]. Together, these observations indicate that Nesprin-1 is important for DNA damage repair, and that the loss of Nesprin-1 leads to an accumulation of damaged DNA.

Pull-down experiments with GST-Nesprin-1 have identified MSH2 and MSH6 as potential binding partners [49]. MSH2 is a DNA mismatch repair and damage recognition protein that forms a complex with MSH6 (MutSα), which binds to DNA mismatches and functions in the repair of DNA double strand breaks [73]. MSH2 and MSH6 expression levels appear to correlate with Nesprin-1. In Huh7 cells with low Nesprin-1 expression levels [49] or human fibroblasts with Nesprin-1 knockdown, the expression levels of MHS2 were low and MSH6 expression was nearly absent. On the other hand, human fibroblasts that express higher levels of Nesprin-1 also express higher levels of MHS2 and MHS6. 

Nesprin-1 is not only important for the expression levels of MHS2 and MHS6, but also for their localization. A key step in recruiting these DNA repair proteins to chromatin is H3K36 methylation [74]. While Nesprin-1 did not affect H3K36 methylation, it was found to be important for the localization of MSH2 and MSH6 to H3K36me3 chromatin [49]. Taken together, Nesprin-1 appears to play important roles in DNA repair. Because persistent DNA damage is one of the key drivers of senescence, the loss of Nesprin-1 promotes senescence [49].

### 2.2. Nesprin-2

Nesprin-2 is another ONM protein that is similar to Nesprin-1 in several functional aspects. Beside its interaction with the microtubules, Nesprin-2 interacts with the actin cytoskeleton and, thus, helps in maintaining the nuclear structure, location and mechanotransduction. Mutations in Nesprin-2 have been implicated in muscular dystrophy, cardiomyopathy and progeria [75,76,77].

One of the cellular changes in senescent cells is a pronounced alteration in mitochondrial function and subcellular distribution [78,79]. A role for Nesprin-2 in senescence-associated mitochondrial function/distribution was suggested from studies in aged pluripotent stem cells (iPSCs) undergoing replicative senescence [51]. While iPSCs are generally accepted to have infinite proliferative capacity, the prolonged culture of iPSCs causes changes in mitochondrial numbers and functionality, as well as iPSC differentiation potential, suggesting that these cells could be a useful model of aging and senescence [80]. Several nuclear envelope changes were observed in aged iPSCs, including changes in Nesprin-2. Specifically, aged-iPSCs showed an increase in Nesprin-2 expression, compared to young iPSCs [51]. The aged iPSCs also showed an abnormal accumulation of mitochondria around the nuclear envelope [51]. Nesprin-2 is known to play a role in mitochondria distribution, with some Nesprin-2 isoforms localize to the mitochondria [81]. Therefore, while direct proof of this function is missing, it is possible that Nesprin-2 contributes to senescence by regulating mitochondrial localization and function.

In addition to a potential role in regulating senescence-associated mitochondrial changes, Nesprin-2 has also been shown to contribute to the DNA damage response that drives replicative senescence. Telomeres play an important role in protecting the chromosome ends [82]. The replication-dependent telomere shortening leads to exposed chromosome ends that drive a DNA damage response, which leads to replicative senescence [83,84,85]. In vascular smooth muscle cells (VSMCs), Nesprin-2 was shown to be important for the DNA damage response in cells undergoing replicative senescence [50]. Nesprin-2 has many isoforms, including isoforms that lack the KASH domain and are, therefore, not membrane-bound [60]. The Nesprin-2 isoform contributing to replicative senescence in VSMC was Nesprin-2β∆KASH1, a KASH-less isoform [50].

Nesprin-2β∆KASH1 interacts with ERK1/2 and PML in the nucleus, forming an Nesprin-2/ERK/PML complex [64]. Furthermore, Nesprin-2β∆KASH1 appears to be an important regulator of nuclear ERK1/2 activity, reducing nuclear ERK activity towards some targets through the sequestration into PML bodies [64]. A knockdown of Nesprin-2 resulted in the increased activation of those ERK targets and led to increased cell proliferation [64].

In the context of proliferative senescence in VSMCs, Nesprin-2β∆KASH1-dependent sequestration of ERK1/2 into PML nuclear bodies was found to be important for the localization of the DNA damage response protein ATM to these PML bodies. The ATM kinase is critical for mediating DNA repair in response to DNA damage and through this function plays an important role in senescence [86,87,88]. Not only does Nesprin-2β∆KASH1 bind ATM, but it helps to localize the ATM-containing complex to sites of DNA damage. Nesprin-2 knockdown leads to a loss of ATM from DNA repair foci and impairment of downstream DNA repair, leading to increased γH2AX levels in Nesprin-2-knockdown cells [50]. This role of Nesprin-2 in the DNA damage response pathway was confirmed by treating proliferative VSMCs with doxorubicin, a DNA damage inducer. Doxorubicin treatment resulted in an increase in phosphorylated ERK1/2 levels and at the same time, an increase in Nesprin-2β∆KASH1. Nesprin-2 seems to be important not only for regulating ATM activity but also to activate the G2/M checkpoint. Nesprin-2 depletion by knockdown experiments lead to the disruption of the Nesprin-2/ERK/PML complex and showed a significant increase in cells with mitotic defects (i.e., telophase bridges and lagging chromosomes) and a 2-fold increase in cells exhibiting micronuclei, both indicative of a failure to fully activate ATM and the ATM-dependent G2/M checkpoint [50]. The Nesprin-2β∆KASH1 interaction with ERK1/2 is, therefore, important for the efficient activation of ATM and the G2/M checkpoint [64,89].

Taken together, Nesprin-2 is a key scaffold for ERK1/2, PML and ATM that brings ATM to the sites of DNA damage and is essential for efficient DNA repair. At the same time, Nesprin-2β∆KASH1 inhibits ERK1/2 activity to prevent cell cycle progression. Therefore, the loss of Nesprin-2β∆KASH1 function leads not only to a loss of ATM from sites of DNA damage, but also to a loss of G2/M checkpoint functions, effectively acting as a double-edged sword, driving cells to senescence [50,64].

Nesprin-2 has also been suggested to play a role in Hutchinson–Gilford progeria syndrome (HGPS). HGPS is an extremely rare disease caused by a de novo mutation that activates a cryptic splice site, resulting in a truncated version of pre-Lamin A that is not processed correctly in the post-translational cleavage steps. This results in the production of a new protein called progerin [38,90]. The accumulation of progerin at the nuclear lamina triggers a number of HGPS-associated cellular phenotypes, including nuclear blebbing, decrease in DNA repair and increase in senescence [91]. Accumulation of progerin in the nuclear lamina interferes with NE protein functions and nucleo-cytoplasmic signaling, including signaling via the canonical WNT/β-catenin pathway [37,52,92]. Reduced levels of active β-catenin activity is found in induced pluripotent stem cells (iPSCs) from HGPS patients [93], suggesting that nuclear envelope functionality is important for β-catenin signaling. Indeed, the reduction in β-catenin has been associated with dysregulation in emerin and Nesprin-2 expression levels, which have both been shown to play a role in β-catenin nucleo-cytoplasmic shuttling. Specifically, emerin, which is an INM protein, was shown to promote export of β-catenin, while Nesprin-2, an ONM protein, promoted nuclear import [52,94,95]. In the skin from a HGPS mouse model [96], elevated emerin levels were observed in the nuclear envelope. Dermal fibroblasts generated from the same animals showed a decrease in Nesprin-2 in the nucleus and nuclear rim [52]. Therefore, low levels of Nesprin-2 coupled with elevated levels of emerin converge in HGPS to promote the loss of β-catenin from the nuclear space. Nuclear β-catenin is required for the upregulation of key target genes, many of which are important for cell proliferation and survival [97,98]. Thus, the loss of Nesprin-2 can contribute to senescence through regulating pro-survival and growth signals.

### 2.3. Nesprin-3

Nesprin-3 is a much smaller KASH-domain protein than Nesprin-1 and Nesprin-2 and it interacts with intermediate filament proteins through the adapter protein plectin. The connection between Nesprin-3 and senescence is currently tenuous and mostly limited to the senescence observed in matrix metalloproteinase (MMP) 14-null mice. MMPs are a group of extra- or pericellular proteases, with important roles in mediating extracellular matrix (ECM) integrity. MMPs have the capability to process essentially all components of the ECM [99,100]. The membrane-bound MMP14 (MT1-MMP) is a member of the collagenases that cleave fibrillar collagens and facilitate collagen degradation essential for ECM remodeling. The loss of MMP14 results in an inability to produce a functional ECM [101], *MMP14*-null cells do not invade and consequently, *MMP14*-null mice display a mixture of phenotypes that result in premature death [102,103,104,105,106]. One of the phenotypes of the *MMP14*-null mice is senescence in heart, kidney, and adipose tissue.

Interestingly, the nuclear morphology of *MMP14*-null fibroblasts was profoundly altered, with blebs and herniations of the nuclear lamina, consistent with the phenotypes observed in senescent cells. These nuclear aberrations were associated with increased DNA damage, as indicated by the increased γH2AX foci [54]. At the molecular level, *MMP14*-null fibroblasts displayed an increase in Lamin A, Nesprin-3 and SUN1/2 protein levels when compared with cells from wild-type mice. The immunofluorescent analysis of the cytoskeleton in *MMP14*-null cells revealed marked alterations in the cytoskeletal organization around the nucleus, some of which were similar to the alterations previously reported in Nesprin-3-null cells [54,107]. The perinuclear and nuclear region showed a significant reduction in the numbers of actin fibers and vimentin displayed an irregular distribution, with marked accumulation at one side of the nucleus and absence in other areas. The nuclear region also showed a diminished number of microtubules [54]. These findings suggest that mice lacking MT1-MMP have deficient connectivity between the nuclear envelope and cytoskeleton, which at least in part contribute to the nuclear abnormalities observed in these animals [54]. Interestingly, the treatment of *MMP14*-null mice with all-trans retinoic acid (ATRA) showed a notable reduction in the nuclear alterations, together with a decrease in the number of senescent cells in vivo. ATRA has previously been shown to regulate levels of Lamin A [108] and ATRA-treated *MMP14*-null mice restored not only Lamin A, but also Nesprin-3 and SUN protein expression to levels similar to those of wild-type mice [54]. It is interesting to note that Lamin A-null mice also do not form ECM correctly, and indeed, cells from HGPS patients show a down-regulation of MMP14 [37,109,110]. Both LaminA-null mice and HGPS patients are characterized by high levels of senescent cells. However, Nesprin-3 was not reported to be affected in Lamin A/C-null mice or HGPS settings. Further work will be necessary to determine the contributions, if any, that Nesprin-3 makes to the senescence phenotype, and if the changes in Nesprin-3 are due to loss of MMP14 or indeed due to senescence.

### 2.4. SUN1/SUN2

Two LINC proteins located in the INM are SUN1 and SUN2, both of which interact with KASH-domain proteins and are essential for the proper NE localization of KASH-domain proteins [111,112]. SUN1 is essential for telomere tethering and homologous recombination in meiosis and SUN1 knockout mice are sterile [113,114]. Because of their essential role in anchoring KASH-domain proteins to the ONM, SUN1/2 play a key role in nuclear migration and anchorage, as well as in mechanotransduction. [113,115,116,117]. Both SUN1 and SUN 2 have also been shown to play a role in senescence via their roles in DNA damage response and repair pathways [53,55,56].

HGPS patient-derived fibroblasts and mouse models of premature aging exhibit an abnormal accumulation of SUN1. Several mouse models mimic some of the pathophysiologies associated with premature aging [118,119,120,121]. In most of these models, senescence is a prominent cellular phenotype [37,122,123]. SUN1 accumulates at the Golgi in MEFs from *Lmna*-null and *LmnaΔ9* mutant mice [12,55]. The accumulation of SUN1 was also observed in fibroblasts from HGPS patients, although no accumulation at the Golgi was observed [55]. When SUN1 was genetically ablated from the *Lmna*-null and *LmnaΔ9* mice, many of the progeric phenotypes were dramatically improved. *LmnaΔ9 SUN1*^−/−^ had increased body weight and longevity compared to *LmnaΔ9*. *Lmna/SUN1* double-null animals showed dramatic improvements in the lordokyphotic phenotype, as well as cardiac and skeletal muscle defects. [55]. At the cellular level, *Lmna/SUN1* double-null and *LmnaΔ9 SUN1*^−/−^ fibroblasts showed improved proliferative capacity compared to their *Lmna*-null and *LmnaΔ9* counterparts. In HGPS-derived fibroblasts, the knockdown of SUN1 dramatically decreased the levels of senescence to levels observed in wild-type fibroblasts. Clearly, SUN1 plays a key role in driving some of the premature aging phenotypes, including senescence. The effects of SUN1 accumulation could be potentially due to cytotoxic Golgi accumulation [55], or it could be due to a role of SUN1 in the DNA response pathway [53].

The SUN1 accumulation observed in HGPS cells appears to be due to reduced protein turnover, since the SUN1 mRNA levels did not differ significantly in the HGPS or normal cells. Increased SUN1 promoted larger and more severely deformed nuclei, an increase in SA β-gal activity and a decrease in cells’ proliferative ability. When SUN1 expression was knocked down by siRNA, it lowered the number of cells with aberrant nuclei compared to the controls. By contrast, when SUN1 was ectopically expressed, it significantly increased the abnormal nuclei. [55]. SUN1 colocalized with progerin in the abnormal nuclear folds and blebs and promoted aberrations in the structure of the membrane, as well as ER network abnormalities [56]. The interaction between SUN1 and progerin (Δ150Lamin A; pre-Lamin A isoform) appears to be enhanced by the farnesylation of progerin.

ER [124] and Golgi [125] morphology and functional abnormalities are known features of senescent cells. HGPS cells showed an accumulation of calnexin (ER marker) and SUN1 at the nuclear envelope, with a disorganized ER network in the cytoplasm and a significant increase in the diameter of the ER lumen. These abnormalities were reduced when SUN1 was depleted [56]. Moreover, the Golgi cisternae were dispersed in HGPS cells expressing high levels of SUN1 [56].

Aside from their role in mediating some of the ER and Golgi senescence phenotypes, SUN proteins are also important in the DNA damage response and repair pathways [53], which are important for senescence, since the accumulation of DNA damage is a key trigger of senescence [70,71]. Treating *SUN1*^−/−^*SUN2*^−/−^ MEFs with methyl methane-sulfonate, a known DNA-damage inducer, resulted in significantly higher DNA fragmentation in the double-null MEFs than in the wild-type MEFs [53,126]. *SUN1*^−/−^*SUN2*^−/−^ MEFs showed reduced γ-H2A.X expression and reduced activation of ATM and Chk1 in response to DNA damage, suggesting an impaired activation of DNA damage response [53]. Because of this reduced capacity to respond to the DNA damage, *SUN1*^−/−^*SUN2*^−/−^ MEFs are more susceptible to DNA damage [53]. At least part of this decreased capacity in the DNA damage response (DDR) may be due to loss of SUN2. SUN2 can interact with DNA-PKcs, which are known to have a critical role in the DDR [53,127,128].

The above findings were reported in MEFs. In human cancer cells, radiation-induced senescence in MCF7 and U2OS cells also brought about changes in SUN1 and SUN2. SUN1 and SUN2 localize away from the nuclear envelope and accumulate in cytosolic aggregates in response to radiation [69]. A decrease in the full-length SUN2 protein level with generation of smaller isoforms was also reported [69].

These observations indicate that the SUN proteins play important roles in recognition and repair of DNA damage. SUN proteins are lost from the nuclear envelope in response to senescence-inducing DNA damage. In addition, SUN proteins play a key role in mediating some of the senescence-associated changes observed in the subcellular structure and organization.

## 3. The LINC Complex, Mechanical Stress and Senescence

Living tissues are continuously exposed to mechanical stress. There are three main types of mechanical stress—tension, compression and shear stress, all of which can cause cell and tissue deformation, including stretching, compaction and bending [129]. Mechanical forces can contribute to disease mechanisms and have been shown to promote the process of senescence [130]. For example, fibroblasts subjected to mechanical strain application showed mitochondrial vacuolization, cytoskeleton depolymerization and increased senescence [130]. Another example can be observed in nucleus pulposus (NP) cells. NP cells are important for forming the cushion between intervertebral discs, are routinely exposed to physiological levels of mechanical pressure, and are a model for studying disc degeneration [131]. NP cells entered premature senescence when exposed to prolonged forces of unphysiological magnitude, either strain or compression [131,132]. Cyclic mechanical tension in NP cells triggers a significant increase SA-β-gal positive cells [131,132], coupled with an increase in DNA damage response proteins, including upregulation in *p*53 and *p*21*^CIP1/WAF1^* expression [131].

Extracellular forces are first sensed by cell surface-adhesion receptors, such as integrins and cadherins. The forces are then guided along cytoskeletal filaments, which transfer the force through the LINC complex to the nucleoplasm [133]. The LINC complex is essential for mechanotransduction (the conversion of mechanical stimuli into biochemical signals), as the transmission of force into the nucleus causes changes in chromatin organization, transcriptional activity, and protein modifications. Not surprisingly, mechanotransduction plays a vital role in cell and tissue differentiation and maintenance [134,135,136]. The LINC complex forms the mechanical link between the cytoskeleton and the nucleoskeleton and, thus, is essential for the force transmission between the ECM and nucleus. Mechanical forces play a key role in HGPS, where patients die of heart attacks or stroke [137]. These patients also often display a loss of smooth muscle cells (SMCs) [138,139,140,141]. To understand the contribution of mechanical stress to this SMC loss, aortic smooth muscle cells (SMC) were isolated from a HGPS mouse model that were transgenic for the progerin protein [142]. These SMCs were cultured on flexible polydimethylsiloxane (PDMS) membranes and exposed to repetitive biaxial stretching for 24 h. Approximately 40% of the progerin-expressing cells died after biaxial strain. No such cell death was observed in SMCs from wild-type mice [141]. This is in line with previous reports that showed that the expression of progerin sensitizes cells to mechanical stress [143,144]. To determine the role of the LINC complex, a Nesprin-2 KASH domain was ectopically expressed. This KASH domain saturates all the SUN binding sites and acts as a dominant negative, disrupting all functional LINC complexes [145]. Indeed, the disruption of the LINC complex increases cell survival by reducing force transmission to the nucleus of progerin-expressing SMCs exposed to repetitive stretching. The amount of DNA damage sustained by the stretched SMCs was also significantly reduced when the dominant negative KASH construct was expressed [141]. Therefore, a functional LINC complex contributes to at least some of the DNA damage observed in cells from HGPS patients and these complexes are likely contributors to the increased levels of senescence observed in these patients.

## 4. Concluding Remarks

**Figure 2:**
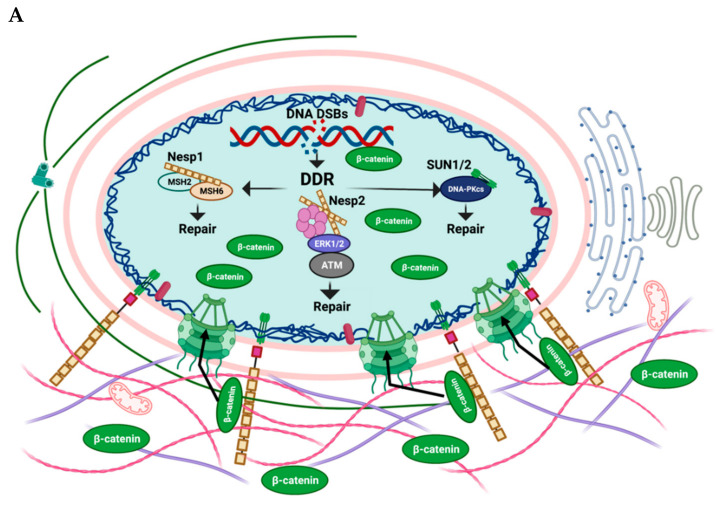

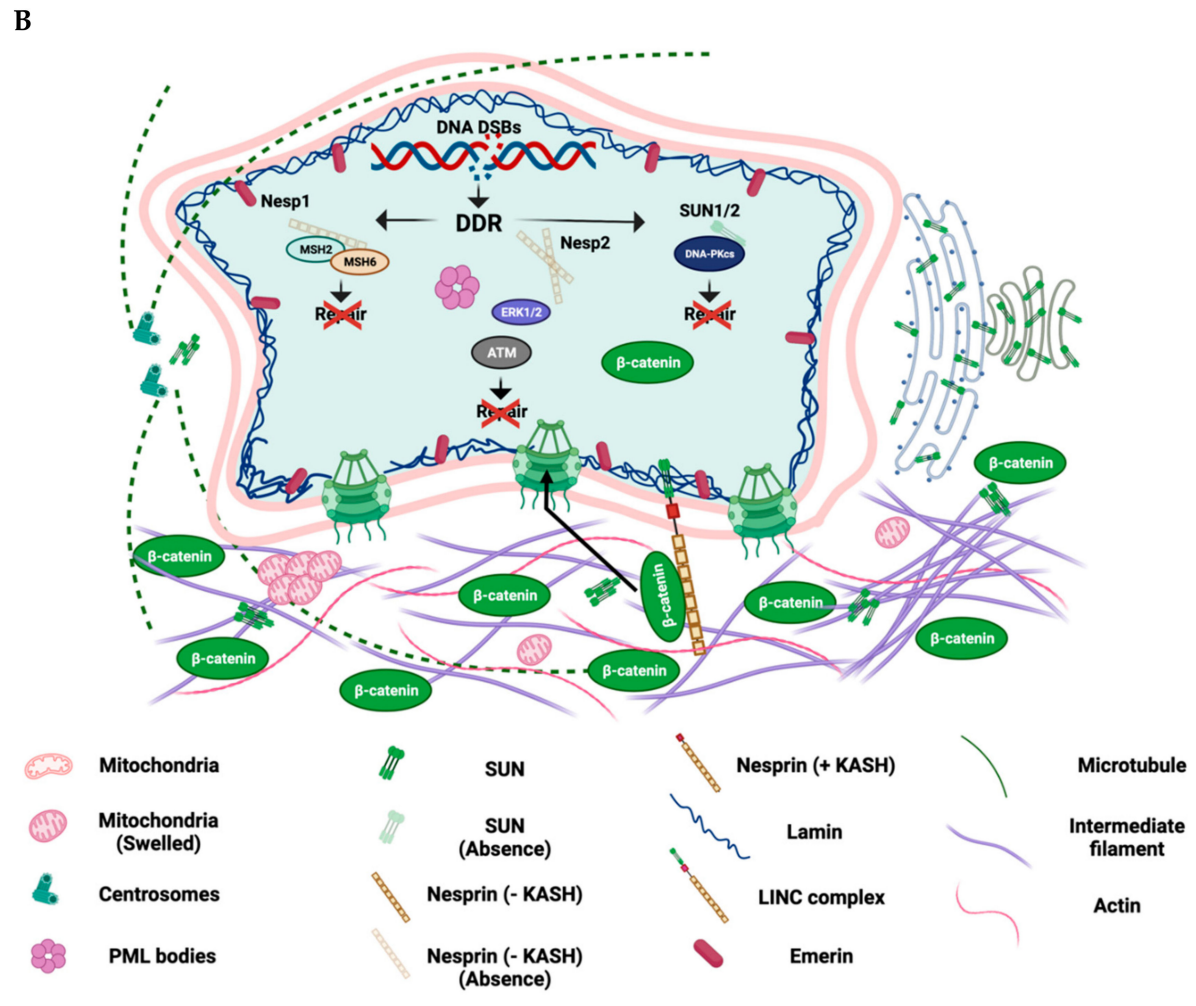
Diagram representing the role of LINC proteins in (**A**) non-senescent and (**B**) senescent cells. (**A**) In non-senescent cells, the mitochondria and cytoskeleton are intact and distributed normally. The nucleus shows a normal shape and size with Lamins lining the inner nuclear membrane and the SUN protein connecting to Nesprins via KASH domains in the PNS. Importantly, DNA damage is repaired through several mechanisms. Three mechanisms that involve LINC complex proteins are shown. (**B**) In senescent cells, mis-localization and aggregation of swelled mitochondria near the nuclear envelope (NE) and an irregular nuclear shape are associated with changes in Nesprins. Loss of Nesprins and SUN proteins lead to an accumulation of DNA damage. Loss of Nesprin-2 leads to a loss of β-catenin from the nucleus. In addition, SUN proteins form cytoplasmic aggregates, can accumulate in the Golgi and can promote ER distension. This figure was created with BioRender.com (accessed on 20 May 2022).

Senescence is part of many physiological and pathophysiological processes. In this review, we highlighted some of the contributions of the LINC complex proteins in senescence. We have reviewed several functions of Nesprins and SUN proteins and discussed their contribution to various senescence-associated processes, including the DNA damage repair pathway (Figure 2). Several of the Nesprin functions are independent of an assembled LINC complex and are affected by Nesprin isoforms that lack the KASH domain. Nesprin-1 interacts with DNA repair machinery and helps to promote an intact genome, promoting cell survival. Nesprin-2β∆KASH1 is essential for the localization of ATM and ERK1/2 to PML nuclear bodies. Furthermore, this complex is localized to sites of DNA damage in a Nesprin-2-dependent manner, promoting the efficient repair of DNA lesions. Nesprin-2 also plays a role in promoting β-catenin nuclear accumulation, an important event for cell proliferation and survival. Finally, SUN1/2 contribute to DNA repair, and the loss of SUN1/2 leads to an accumulation of DNA damage. Thus, Nesprins and SUN-domain proteins contribute to the development or avoidance of senescence by modulating DNA repair. Senescence is also associated with several cellular changes, such as loss of mitochondrial function, enlargement of the nucleus and lobulations of the nuclear membrane. Nesprins appear to be involved in these aspects of senescence to varying degrees and we have highlighted the contributions of various Nesprins to these senescence-associated changes. Finally, an intact LINC complex can lead to increased DNA damage in already diseased tissues and organs and LINC complexes can contribute to senescence through mechanotransduction. As senescence continues to gain attention as a major contributing factor to aging and disease, we believe that additional functions for LINC complexes and LINC complex proteins will be uncovered in regulating senescence and senescence-associated phenotypes.

## Figures and Tables

**Table 1:** Summary of LINC complex proteins in senescence.

LINC Protein	Model/Cell Line	Mode of Senescence Induction	Cellular Mechanisms/Structures Affected	Ref’s
Nesprin-1	Embryonic mouse mesenchymal stem cell line (CH310T1/2)	Nesprin-1 KD	Increase (↑) DNA damage (DSBs) due to DNA mismatch repair (MMR) impairment • Increase in the mean centrosome-nucleus distance • More than two centrosomes ↑ SUN1 & SUN2 (protein)	[48,49]
	Human dermal fibroblasts (HFs)	Nesprin-1 KD Etoposide, Hydroxyurea (HU) and UV	** Knockdown **: Increase in the mean centrosome-nucleus distance More than two centrosomes Nesprin-1 KD affects the interaction of Nesprin-1 actin binding domain (ABD) to the DNA mismatch repair and damage recognition protein MSH2. ** Treatments **: Decrease (↓) MMR (etoposide) ↑ DNA damage (all treatments) ↓ Ku70 (HU treatment) • Nearly no MSH6 mRNA	
	Human colorectal carcinoma cell line (DLD-1). These cells are Nesprin-1 deficient.	N/A	** Compared to normal human fibroblast **: • Abnormal nuclear morphology, centrosomal aberrations • Deficient in DNA mismatch repair ↓ MSH2 mRNA • Nearly no MSH6 mRNA/protein	
	Human hepatocellular carcinoma cell lines (Hep3B, Huh7) These cells have low endogenous Nesprin-1 expression.	Etoposide, Hydroxyurea (HU) and UV	**Non-Treated (comparing Hep3B and Huh7 to normal liver cells)** ↑ SUN1 & SUN2 (mRNA) ↑ SUN1 (protein) • Nuclear shape defects (folds, lobulations, blebs, and micronuclei) ↑ Centrosome-nucleus distance & numbers **Treatments**: ↑ DNA damage ↓ Ku70 (HU & UV treatments) ↓ MSH2 mRNA • Nearly no MSH6 mRNA/protein	
Nesprin-2 variant (β∆KASH1)	Human aortic vascular smooth muscle cells (VSMCs)	Population doublings (PDs)	**Presenescence**: ↑ Numbers of PML NBs per nuclei & size **Presenescence & senescence**: ↑ DNA damage-mediated p53 signaling • ATM mislocalization away from DNA repair foci • Impairment of downstream DNA repair signaling ↑ Nesprin-2/ERK2/promyelocytic leukemia protein nuclear bodies (PML NB) localization in presenescence compared to senescence **Senescence**: ↑ Numbers of PML NBs per nuclei ↑ Prelamin A accumulation ↓ Farnesylated protein converting enzyme 1 (FACE1) protein	[50]
		Doxorubicin	↑ Levels of phosphorylated ERK1/2 ↑ Nesprin-2βΔKASH1/ERK2/phosphorylated ERK1/2 association	
		LaminA/C KD+ Doxorubicin	↓ Nesprin-2 and ERK2 co-localization at PML NBs ↑ PML NBs size	
		Zmpste24 KD	**Proliferative:** ↑ ERK2 localization at PML NBs ↑ PrelaminA accumulation	
		Nesprin-2 KD Doxorubicin	**Presenescent**: • Loss of Nesprin-2 from PML NBs ↑ Comet tail intensity ↑ Mitotic defects **Proliferative**: G2/M checkpoint failure: ↑ Micronuclei	
Nesprin-2	Aged-Pluripotent stem cells (a-iPSCs)	iPSCs cultured for more than one year	**mRNA expression levels**: ↑ Nesprin-2, LMNA, NF-kB, emerin ↓ SIRT7 • Nuclear dysmorphisms (shape, blebs and folded NE) • Abnormal accumulation of mitochondria associated to NE alterations • Slow actin polymerization rate	[51]
	HGPS dermal fibroblasts HGPS mice interfollicular epidermis (IFE)	N/A	• Impaired polarity • Impaired Wnt/β-catenin signaling (↑ nuclear export of β-catenin) ↑ Progerin **Postnatal day 4 (mice)**: ↑ Emerin at nuclear rim ↓ Nuclear β-catenin Human HGPS fibroblasts: ↓ Nesprin-2 at nuclear rim	[52]
SUN1/2	SUN1/2^-/-^ MEFs	N/A	↓ Perinuclear heterochromatin compared to wild-type MEFs	[53]
		Hydroxyurea	**Compared to wild type MEFs:** ↑ DNA damage • ATM activation (phosphorylation) failure • SUN1& SUN2 loss affects the SUN proteins/DNA dependent protein kinase catalytic subunit (DNA-PKcs) interaction required for DNA repair & cell proliferation	
		Methyl methane-sulfonate	↑ DNA fragmentation ↑ Prominent comet tails	
	*Mmp14^-/-^* mouse embryonic fibroblasts (MEFs)	N/A	↑ Abnormal nuclear lamina morphology (blebs and herniations) ↑ DNA damage **Perinuclear and nuclear region**: ↓ Actin fibers • Irregular vimentin distribution (accumulation on one side of nucleus) ↓ Tubulin filaments ↑ Nesprin-3 (mRNA, Protein) ↑ SUN1 & SUN2 (mRNA)	[54]
	*LMNA*-/- mouse embryonic fibroblasts (MEFs)	N/A	Irregular nuclear shape & blebs formation Frequent nuclear herniations and blebs Accumulation of SUN1 at Golgi SUN1 accumulation due to decrease in protein turnover No change in SUN2 (level/distribution) Modest ↑ in emerin & Nesprin-1 ER localization	[55]
	Lmna∆9 mutant MEFs	N/A	Irregularly shaped nucleus with frequent herniations and blebs that were reduced in LMNA/SUN1 DKO MEFs	
	Hutchinson-Gilford progeria syndrome (HGPS) human skin fibroblasts	N/A	**Compared to normal human fibroblast**: ↑ SUN1- levels • Cells exhibited larger nuclei and more severe nuclear morphological distortions • SUN1 KD alleviated the abnormalities • SUN1 overexpression (exogenous) augmented abnormalities ↓ Chromatin disorganization and heterochromatin loss↓ Pan heterochromatin marker histone H3K9me3	
	Hutchinson-Gilford progeria syndrome (HGPS) human skin fibroblasts	N/A	** Structural changes: ** • Nuclear envelope (NE) • Endoplasmic reticulum (ER) network disorganization ↑ ER lumen diameter • Stacked multiple membrane bilayers, forming vesicles or invaginations at the nuclear periphery • Dispersed Golgi cisternae • Nuclear pore complex clustering (SUN1 dependent) ↑ Accumulation of SUN1	[56]
	HeLa cells	Progerin overexpression	↑ Perturbed nuclear morphology (∼80% of cells) • Reorganized ER network	
		Progerin overexpression & SUN1 KD	↓ Nuclear and cellular aberrancies ↓ ER network abnormalities	

## Data Availability

Not applicable.
